# Characterization of eyes, photoreceptors, and opsins in developmental stages of the arrow worm *Spadella cephaloptera* (Chaetognatha)

**DOI:** 10.1002/jez.b.23193

**Published:** 2023-02-28

**Authors:** Tim Wollesen, Sonia V. Rodriguez Monje, Adam P. Oel, Detlev Arendt

**Affiliations:** ^1^ Department of Evolutionary Biology, Faculty of Life Sciences University of Vienna Vienna Austria; ^2^ Developmental Biology Unit European Molecular Biology Laboratory Heidelberg Germany

**Keywords:** cryptochrome, eye evolution, Gnathifera, Lophotrochozoa, Spiralia, xenopsin

## Abstract

The phylogenetic position of chaetognaths, or arrow worms, has been debated for decades, however recently they have been grouped into the Gnathifera, a sister clade to all other Spiralia. Chaetognath photoreceptor cells are anatomically unique by exhibiting a highly modified cilium and are arranged differently in the eyes of the various species. Studies investigating eye development and underlying gene regulatory networks are so far missing. To gain insights into the development and the molecular toolkit of chaetognath photoreceptors and eyes a new transcriptome of the epibenthic species *Spadella cephaloptera* was searched for opsins. Our screen revealed two copies of *xenopsin* and a single copy of *peropsin*. Gene expression analyses demonstrated that only *xenopsin1* is expressed in photoreceptor cells of the developing lateral eyes. Adults likewise exhibit two *xenopsin1* + photoreceptor cells in each of their lateral eyes. Beyond that, a single *cryptochrome* gene was uncovered and found to be expressed in photoreceptor cells of the lateral developing eye. In addition, *cryptochrome* is also expressed in the cerebral ganglia in a region in which also *peropsin* expression was observed. This condition is reminiscent of a nonvisual photoreceptive zone in the apical nervous system of the annelid *Platynereis dumerilii* that performs circadian entrainment and melatonin release. *Cryptochrome* is also expressed in cells of the corona ciliata, an organ in the posterior dorsal head region, indicating a role in circadian entrainment. Our study highlights the importance of the Gnathifera for unraveling the evolution of photoreceptors and eyes in Spiralia and Bilateria.

## Highlights

‐ As light‐sensitive molecules two *xenopsins*, a *peropsin* and a *cryptochrome* are present in developmental stages and adults of the chaetognath *Spadella cephaloptera*.
‐ *Xenopsin1* appears to be the only opsin expressed in the chaetognath eyes.
‐ *Crypotochrome* and *peropsin* are expressed in the cerebral ganglia indicating a role in circadian entrainment.

## Graphical abstract

Adult eye anatomy of the chaetognath *Spadella cephaloptera*. The spherical shaped adult eyes exhibit photoreceptors with distal photoreceptive segments organized into four tightly packed domains. As light‐sensitive molecules two xenopsins, a peropsin and a cryptochrome are present in developmental stages and adults of the chaetognath *S. cephaloptera*. The xenopsins appear to be the only opsins expressed in the chaetognath eyes. Crypotochrome is coexpressed with peropsin in the cerebral ganglia indicating a role in circadian entrainment.

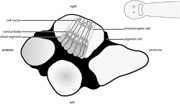

**Abbreviations**
BCIP: 5‐brom‐4‐chlor‐3‐indoxylphosphat
BLAST: basic local alignment search tool
cDNA: complementary deoxyribonucleic acid
DIG: digoxigenin
NBT: nitro blue tetrazolium
NCBI: National center for biotechnology information
PBT: phosphate buffered saline with tritonX‐100
PCR: polymerase chain reaction
Sce: *Spadella cephaloptera*

## 1 INTRODUCTION

Darwin was puzzled by the organizational principles of complex eyes and admitted that it was difficult to view these organs as products of natural selection (Darwin, 1859). Ever since then scientists have been intrigued by the organization and evolution of eyes which were assumed to be lost and gained multiple times independently (Salvini‐Plawen & Mayr, 1977). There is, however, evidence that simple cup‐shaped eyes with photoreceptor cells and shading pigments already existed in the last common bilaterian ancestor (Arendt et al., 2004). While rhabdomeric photoreceptors store photopigments (opsins) in the expanded apical surface folded into microvilli, ciliary photoreceptors employ surface‐extended cilia for the same purpose (Arendt et al., 2004). For a long time, the task of vision was thought to be carried out exclusively by rhabdomeric photoreceptors in invertebrates, and by ciliary photoreceptors in vertebrates (Eakin, 1982). Recent studies, however, showed that photoreceptors may also co‐express different types of opsins and that distinguishing photoreceptors on morphological grounds is not as clear‐cut as previously assumed (Arendt, 2017; Vöcking et al., 2017). Combined molecular and morphological studies provide a survey of eye and photoreceptor organization and of opsins in diverse bilaterian evolutionary lineages (Arendt & Wittbrodt, 2001; Randel et al., 2013; Wollesen et al., 2019). Together, available work suggests that the last common bilaterian ancestor possessed several opsins such as a ciliary opsin (c‐opsin), a canonical rhabdomeric opsin (r‐opsin), a noncanonical r‐opsin, a go‐opsin, a neuropsin, a retinal pigment epithelium‐retinal G protein‐coupled receptor/peropsin/retinochrome, and, possibly, a xenopsin (Ramirez et al., 2016).

**Figure 1:**
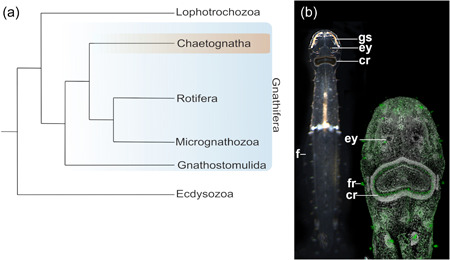
Chaetognaths and their phylogenetic position. (a) Chaetognatha belongs to the Gnathifera, a taxon being sister to the Lophotrochozoa. The latter includes taxa such as Mollusca, Annelida or platyhelminths and composes together with Gnathifera the Spiralia *sensu* Marlétaz et al. (2019). (b) An adult of the chaetognath *Spadella cephaloptera* (dorsal view and anterior faces up; individual ~4 mm in length) (left). Confocal laserscan of the head and anterior trunk region of an adult *S. cephaloptera* (green: tyrosinated tubulin; grey: cell nuclei/DAPI). As torpedo‐shaped predators chaetognaths are equipped with cuticular grasping spines (gs), and horizontally extending fins (f). As sensory organs they possess a pair of compound eyes (ey), numerous fence receptors, and a ring of ciliated cells (“corona ciliata” [cr]). DAPI, 4′,6‐diamidino‐2‐phenylindole.

Bilaterian animals are subdivided into Deuterostomia and Protostomia, and the latter comprise Ecdysozoa (with arthropods, nematodes, and many other molting animals) and Spiralia, a large assembly of mostly marine invertebrates. In Spiralia, the expression of opsins and other photoreceptor genes has so far been studied in mollusks (Vöcking et al., 2015, 2017; Wollesen et al., 2019), annelids (Arendt et al., 2004), platyhelminths (Rawlinson et al., 2019), and brachiopods (Passamaneck & Martindale, 2013). Gnathifera have recently been identified as the sister taxon to all other Lophotrochozoa (Marlétaz et al., 2019). They comprise the arrow worms (Chaetognatha), wheel animals (Rotifera), jaw worms (Gnathostomulida), and the tiny Micrognathozoa and have not yet been investigated with respect to genes underlying photoreceptor formation and function (Figure 1; Marlétaz et al., 2019). Chaetognaths are a rather small taxon of marine, torpedo‐shaped coelomic animals with horizontally projecting fins, and cuticular grasping spines to catch prey (Shinn, 1997). As a major macroplanktonic component without respiratory or circulatory systems, chaetognaths exhibit traits reminiscent of deuterostomes (e.g., aspects of the gastrulation process) as well as of protostomes (e.g., nervous system development). Hence, their phylogenetic position had been contentious until phylogenetic analyses recently placed them within the Gnathifera (summarized by Harzsch & Wanninger, 2010; Harzsch et al., 2015; Marlétaz et al., 2019).

Most chaetognath species possess a pair of subepidermal eyes that are encapsulated by extracellular matrix and sheath cells on the dorsal side of the head and that are linked to the cerebral ganglia via optic nerves (Figure 1b; Eakin & Westfall, 1964; Goto & Yoshida, 1984; Müller et al., 2019). In these eyes, photoreceptor cells either present the photoreceptive membranes directly to incoming light, or sequester the receptive membranes deeper within the eye such that light passes first through the main cell body of the photoreceptor cell. The inverted eye is a spherical dorso‐ventrally flattened structure with a pigment cell in the center. Its photoreceptor cells are composed of a distal segment, representing a modified cilium, that connects to the proximal segment via a conical body and ends in an axon (Eakin & Westfall, 1964). The conical body is unique to chaetognaths and exhibits refractive properties.

Evidence from histochemistry and peak spectral analysis suggest that a rhodopsin‐like pigment is present in the distal segment in *Paraspadella gotoi* (Goto & Yoshida, 1988; Sweatt & Forward, 1985). The proximal segment, which forms part of the cell body, exhibits a brush of microvilli and thus may also be photosensitive (Eakin & Westfall, 1964). It is as yet unclear which type of opsin is expressed in the distal and/or proximal photoreceptor segment in chaetognaths. As in vertebrates the photoreceptor cell bodies send out an axon where the light penetrates the cell (Eakin & Westfall, 1964). A single chaetognath eye may be composed of 70–600 photoreceptor cells (Goto & Yoshida, 1984). In the “everted” eye type, the distal photoreceptive process of each photoreceptor cell points to the periphery and in most of the species investigated more than one pigment cell is present (Goto & Yoshida, 1984). Besides their eyes, chaetognaths are also equipped with several other sensory organs such as the ciliary fence receptors or the corona ciliata, a ring of ciliated and unciliated cells that is located in the dorsoposterior head region (Müller et al., 2014). Various roles that have been attributed to the corona ciliata include excretory, secretory, or (chemo)sensory function. Ciliary fence receptors have been shown to detect hydrodynamic stimuli and react to close‐range mechanosensory input (attack or escape movements) (Bone & Goto, 1991; Feigenbaum & Maris, 1984; Feigenbaum & Reeve, 1984; Horridge & Boulton, 1967).

In the present study, we investigated the presence of opsins and other light‐sensitive proteins in developmental stages and adults of the epibenthic chaetognath *Spadella cephaloptera*. This species possesses inverted photoreceptors and is probably the best investigated chaetognath with respect to its nervous and sensory systems (summarized in Harzsch et al., 2015; Müller et al., 2019). To this end, we searched for homologs of opsins and other light‐sensitive proteins and identified two copies of *xenopsin* and single‐copy orthologs of *peropsin* as well as *cryoptochrome*. *Peropsin* orthologs have been found in all major bilaterian groups (Sun et al., 1997), while *xenopsin* is only present in Spiralia (and possibly in Cnidaria; Arendt, 2017; Ramirez et al., 2016). Cryptochrome is a key player of the circadian system in animals and plays a role in light sensing as well as entrainment of the circadian oscillator (Emery et al., 1998; Mei & Dvornyk, 2015; Thresher et al., 1998). In our study, we demonstrate that *xenopsin1* likely represents the only opsin expressed in the chaetognath eyes, together with *cryptochrome*, which is also expressed in the corona ciliata.

Given that *xenopsin* appears to have been lost in most major bilaterian lineages, yet is preserved in several spiralian lineages, it has been difficult to infer the ancestral physiological role for *xenopsin*‐mediated photoreception. Our work implies *xenopsin*‐mediated vision in at least one spiralian clade, and highlights the value of more studies on the molecular underpinnings of sensory biology in this important and diverse phylum.

## 2 MATERIALS AND METHODS

### 2.1 Ethics, collection, and culture of animals

Individuals of the chaetognath *S. cephaloptera* (Busch, 1851) were collected in front of the Station Biologique de Roscoff, Roscoff, France in summer 2018 and transferred to an aquarium at the European Molecular Biology Laboratory (EMBL) in Heidelberg. Chaetognaths are hermaphrodites and adults started to reproduce in the aquarium at 18°C water temperature. Adults were kept in petri dishes and fed with artemia. After oviposition adults were transferred to the aquarium. The developing individuals were inspected with a stereo microscope, collected with plastic pipettes, and fixed when needed.

### 2.2 RNA extraction and fixation of animals for in situ hybridization experiments

Adult *S. cephaloptera* were starved for three days and killed together with developmental stages covering early zygotes to hatched one month old juveniles for RNA extraction by using a RNA extraction kit (Qiagen). Additional adults and developmental stages were carefully anesthetized in 7.14% MgCl_2_ before fixation and fixed for 1 h at room temperature for in situ hybridization experiments and treated as previously described (Wollesen et al., 2015). Extracted RNA was used for transcriptome sequencing (see below) and for the synthesis of complementary DNA (cDNA) used for subsequent riboprobe synthesis.

### 2.3 Transcriptome sequencing and assembly

For the transcriptome, pooled total RNA was Illumina 150 bp paired‐end sequenced and resulting in a total of 57,486,532 paired reads. The short‐read libraries were preprocessed using *Trimmomatic* (v. 0.36; Bolger et al., 2014) to remove known specific Illumina adapters from the paired‐end libraries (Illumina universal adapter). Filtering by quality and length was performed with a SLIDINGWINDOW:4:15 MINLEN:36. First and last nucleotides from reads with low quality score were clipped and the library file was converted into fasta format using fq2fa from SeqKit (version 0.11.0). Quality of the initial and filtered library was assessed with the software FastQC (v.0.11.8; Wingett & Andrews, 2018) considering quality score of the bases, GC‐content, and read length. 11.83% of reads were excluded during the preprocessing procedure resulting in a total of 50,686,453 reads. The assemblies and all downstream analyses were conducted with a high‐quality and clean library. The filtered transcriptome was assembled into contiguous cDNA sequences with IDBA_tran v1.1.3 software (Peng et al., 2013) using the default settings (except: −mink 20 −maxk 80 −step5). The resulting assembly was assessed using the tool QUAST (http://quast.bioinf.spbau.ru). The number of contigs was 148,988 with 8.4137 contigs longer than 1000 bp. The number of reconstructed bases was 259.028.025 with 222.358.174 contigs longer than 1000 bp. The length of the largest contig was 35.950,00, the N50 2.179,17, the N75 1.592,00, and the GC content 45.74%. Raw reads obtained by Illumina sequencing as well as the assembled transcriptome are accessible on Zenodo (https://zenodo.org/record/7602960#.Y90U0oSZOUk/DOI:10.5281/zenodo.7602960).

### 2.4 Alignment and phylogenetic analysis

Candidate genes were identified by Protein BLAST searches (Altschul et al., 1990) of orthologous bilaterian amino acid sequences against the transcriptome of *S. cephaloptera* (see above). The phylogenetic analysis was performed for the predicted protein sequences of Sce‐Xenopsin1, Sce‐Xenopsin2, and Sce‐Peropsin building upon the analyses of Vöcking et al. (2021) and Ramirez et al. (2016) (Supporting Information: Figure S1). Orthologous sequences from various metazoan species were retrieved from NCBI (https://www.ncbi.nlm.nih.gov/) and Uniprot (https://www.uniprot.org) for the phylogenetic analysis. Multiple sequence alignment was performed with MAFFT v7.123b (Katoh & Standley, 2013) and the alignment was manually trimmed in AliView v.1.26 (Larsson, 2014). The best‐fitting amino acid replacement model was estimated with Prottest3 v3.4.2 (Darriba et al., 2011). The bayesian phylogenetic analysis on 84 taxa and 308 characters was carried out with MrBayes v3.2.7a in CIPRES Science Gateway (https://www.phylo.org; Miller et al., 2010). The LG + I + G + F amino acid replacement model was estimated with Prottest3 v3.4.2 (Darriba et al., 2011), in addition to 2,760,000 generations. The resulting consensus tree was visualized and adjusted in iTOL v6 (https://itol.embl.de). A Bayesian analysis of cryptochromes was carried out using Ozturk (2017) as reference (Supporting Information: Figure S2). The Bayesian phylogenetic analysis on 37 taxa and 510 characters was carried out with MrBayes on XSEDE v3.2.7a in CIPRES Science Gateway (https://www.phylo.org). The LG G + I amino acid replacement model was estimated with Prottest3 v3.4.2 (Darriba et al., 2011), in addition to 205,000 generations until the average standard deviation of split frequencies got <0.01 (0.009943). The resulting consensus tree was visualized and adjusted in iTOL v4.4.1 (https://itol.embl.de/about.cgi).

### 2.5 Molecular isolation of RNA transcripts

A first‐strand cDNA Synthesis Kit for rt‐PCR (Roche Diagnostics GmbH) was used for first‐strand cDNA synthesis of the RNA pooled from different developmental stages of *S. cephaloptera*. Identified gene sequences in sense orientation were used to design gene‐specific primers with an annealing temperature of >60°C. Reverse primers containing part of the T7 promotor sequence (5′‐TAATACGACTCACTATAGGG‐3′) followed by a reverse complement of the gene specific sequence. Polymerase chain reactions (PCRs) were successfully carried out with the following primer sequences:

*Sce_xenopsin1*

Ffw: GTCGGACTTGTTCATGTGCTCCGTC

Rev: TAATACGACTCACTATAGGGCGTCGCGGTGACGCTCAGTTC

*Sce_xenopsin2*

Ffw: CTGCGGAACATCGCTAGAAACGGTTTG

Rev: TAATACGACTCACTATAGGGGGCTGAAACGCCATTTTTATCCATTCTTGG

*Sce_peropsin*

Ffw: CTGGATCTGGGGAGATCTCGGCTG

Rev: TAATACGACTCACTATAGGGGAGCCTCGGGAAATCGGAGACCAC

*Sce_cryptochrome*

Ffw: GATCCTCGGGAGATCTTTGCTCGTCTC

Rev: TAATACGACTCACTATAGGGGCCAAAGATGACCGCGCGTGAG

Five microliters of each PCR product were size‐fractioned by gel electrophoresis and the remaining 45 µL of the PCR product were cleaned up with a QIAquick PCR Purification Kit (Qiagen) if size estimation and expected sequence length matched. PCR products were sent for sequencing to confirm gene identity and sequences of *Sce‐xenopsin1*, *Sce‐xenopsin2, Sce‐peropsin*, and *Sce‐cryptochrome* were deposited on Genbank (Accession numbers: Sce_xenopsin1 [MN735184), Sce_xenopsin2 [OM687487], Sce_peropsin [MN735185], Sce_cryptochrome [MN735186]).

### 2.6 Probe synthesis and whole‐mount in situ hybridization

In vitro transcription reactions were performed with the above‐mentioned templates, digoxigenin‐UTP (DIG RNA Labeling Kit; Roche Diagnostics), and T7 polymerase (Roche Diagnostics GmbH) for the synthesis of antisense riboprobes, according to the manufacturer's instructions. For whole‐mount in situ hybridization experiments, specimens were rehydrated into PBT (phosphate buffered saline + 0.1% Tween‐20) and treated with Proteinase‐K at 37°C for 10 min (10 µg/mL in PBT). Specimens were prehybridized in hybridization buffer for 4–10 h at 63°C (see Wollesen et al., 2015 for details). Hybridization was performed at the same temperature with probe concentrations ranging between 1 and 2 μg/mL for 21–24 h. A DIG‐labeled AP‐antibody was used at a dilution of 1:2500 in blocking solution at 4°C overnight. Color development in the NBT/BCIP/alkaline phosphatase buffer solution took 6–24 h at 4°C. Some specimens were counterstained with DAPI to visualize cell nuclei (Sigma‐Aldrich). A minimum of 30 individuals per stage were investigated. The majority of whole‐mount preparations were cleared in a solution of 2,2′‐thiodiethanol (Sigma‐Aldrich), mounted on objective slides and analyzed. Preparations were documented with an Olympus BX53 Microscope (Olympus). In addition, developmental stages were scanned with a Leica confocal SP8 microscope (Leica Microsystems) using brightfield, autofluorescence, and reflection mode scans to document the precise cellular location of transcripts (Jékely & Arendt, 2007). If necessary, images were processed with GIMP (version 2.10.10, www.gimp.org) to adjust for contrast and brightness. Sketch drawings were created with Inkscape (version 0.92.4, www.inkscape.org).

## 3 RESULTS

### 3.1 Phylogenetic and sequence analysis

We identified single‐copy orthologs of *xenopsin1, xenopsin2*, and *peropsin* in the transcriptome of *S. cephaloptera*. Their predicted protein sequences cluster well with their bilaterian orthologs in our phylogenetic analysis (Supporting Information: Figure S1). Compared to other bilaterians, the characteristic “NPXXY” motif and tripeptide (“NXQ”) for G‐protein activation of the c‐terminal Sce‐Xenopsin1, Sce‐Xenopsin2, and the Sce‐Peropsin contain slightly different residues, that is, NPLVV + SAR in Sce‐Peropsin and DPILY + NKR in Sce‐Xenopsin1 and Sce‐Xenopsin2 (Passamaneck et al., 2011; Vöcking et al., 2017). The aberrant Sce‐Xenopsin residues are identical to those of the Xenopsin found in the chaetognath *Pterosagitta draco* (Rawlinson et al., 2019). Sce‐Xenopsins and Sce‐Peropsin exhibit both the highly conserved lysine in the retinal binding domain that constitutes the Schiff base with the retinal chromophore forming the photopigment. The sequence of *Sce‐Xenopsin2* could not be amplified by PCR and hence no riboprobe was synthesized for this gene. No other opsins were identified by BLAST searches of bilaterian orthologs against the transcriptome of *S. cephaloptera*. Sce‐Cryptochrome clusters with other type‐1 cryptochromes (Supporting Information: Figure S2).

### 3.2 Gene expression and anatomy of the developing chaetognath eyes

**Figure 2:**
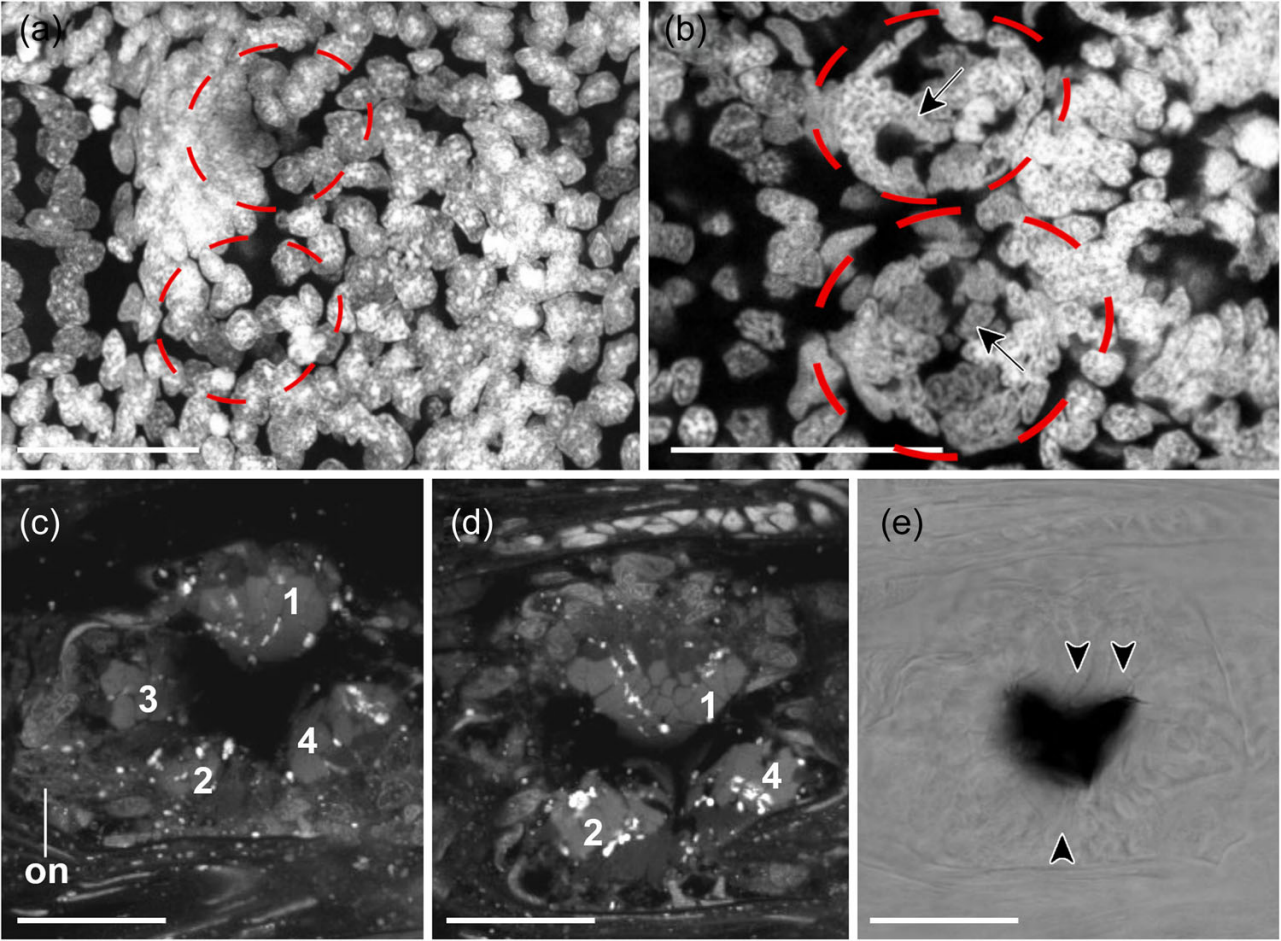
The developing eye of *Spadella cephaloptera*. Dorsal views, anterior to the left. Cell nuclei (DAPI) staining except E (bright field image). (a) The eyes (encircled) of embryos still surrounded by an egg capsule (~20 hpl) appear disorganized with cells not arranged in a circular fashion. (b) In contrast to earlier embryos, recently hatched individuals (~28 hpl) exhibit eyes (encircled) with spherical shape, however, individual cells are still not arranged as seen in subsequent stages (arrow). Eyes do not exhibit pigment cells. (c, d) Adult *S. cephaloptera* exhibit distal segments of photoreceptors that are tightly packed into four packages (1–4). While packages 1 & 2 are located more dorsally, packages 3 & 4 are located more ventrally. The optic nerve (on) connects the eye with the cerebral ganglia (not shown; (Rieger et al., 2011)). (e) The pigment cell (black) is surrounded by photoreceptors (arrowheads). Scale bars: 20 µm. DAPI, 4′,6‐diamidino‐2‐phenylindole.

**Figure 3:**
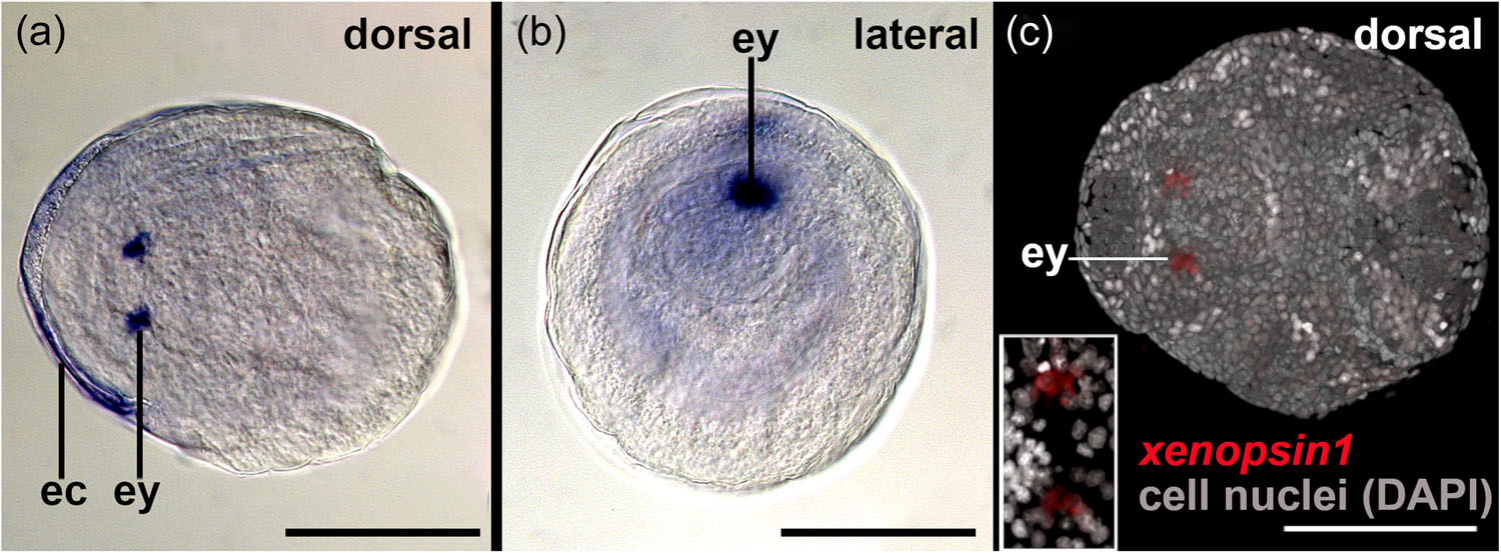
*Xenopsin1* expression in early encapsulated embryos of *Spadella cephaloptera* (~20 hpl). Whole‐mount in situ hybridization, anterior to the left. (a) *Sce‐xenopsin1* + photoreceptor cells in the eyes (ey) of embryos inside their egg capsule (ec). The egg capsule is unspecifically stained. (b) The curled‐up embryo expresses *sce‐xenopsin1* in the developing eyes, in cells that probably correspond to the photoreceptors. (c) The putative photoreceptor cells are not arranged in a circular pattern highlighted by this confocal laser scanning scan. Scale bars: 150 µm.

**Figure 4:**
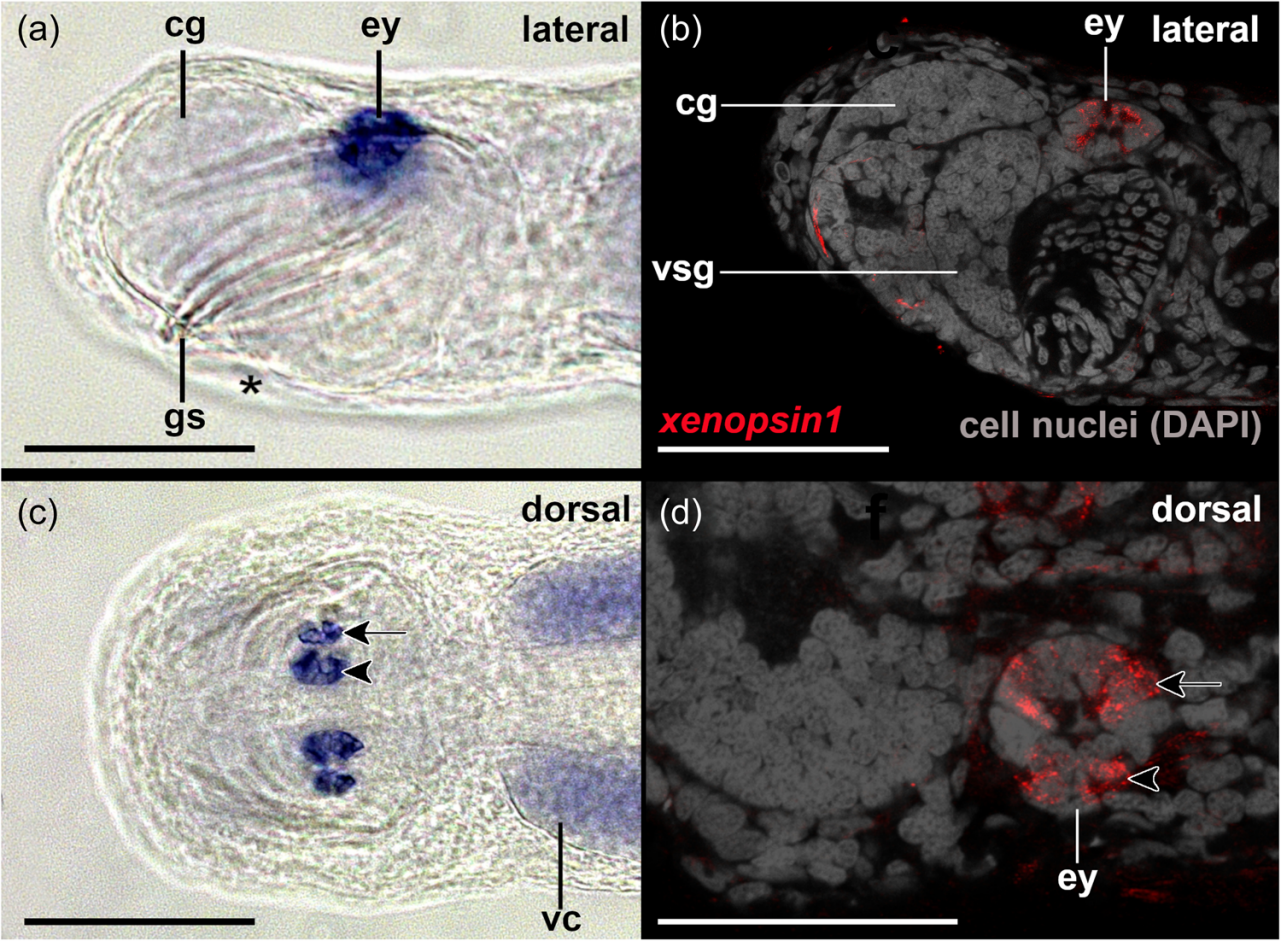
*Xenopsin1* expression in early hatchling juveniles of *Spadella cephaloptera* (~60 hph). Whole‐mount in situ hybridization of the head region, anterior to left. (a) *Sce‐xenopsin1* + photoreceptor cells are located in the lateral regions of the eyes (ey) as also highlighted by confocal laser scanning microscopy in (b). (c) *Xenopsin1*‐expressing photoreceptor cells in the eyes with a close‐up by confocal laser scanning microscopy of the left eye in (d). cg, cerebral ganglion; gs, grasping spines; vc, ventral nerve cord; vsg, vestibular ganglion. Scale bars: 100 µm (except D: 50 µm).

**Figure 5:**
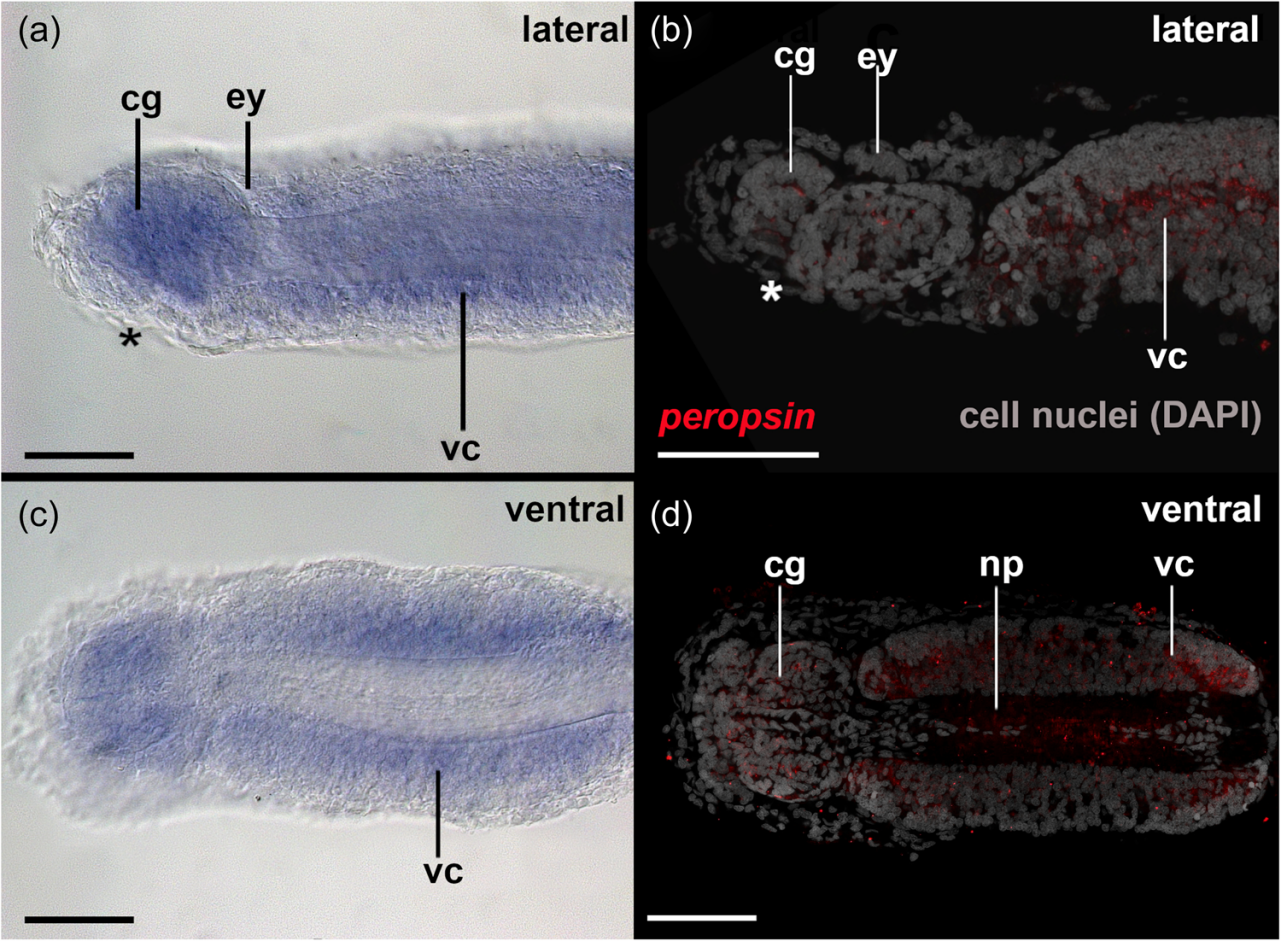
*Peropsin* expression in early hatched juveniles of *Spadella cephaloptera* (~ 24 hph). Whole‐mount in situ hybridization of the head region, anterior to left. (a) *Peropsin* is expressed in the region of the cerebral ganglia (cg) and the ventral nerve cord (vc) but not the eyes (ey) as highlighted by confocal laser scanning microscopy in (b). (c) Ventral view showing *peropsin* + cells in the ventral nerve cord but not the neuropil of the latter as highlighted by confocal laser scanning microscopy in (d). asterisk, mouth; cg, cerebral ganglion; gs, grasping spines; vc, ventral nerve cord; vsg, vestibular ganglion. Scale bars: 100 µm.

**Figure 6:**
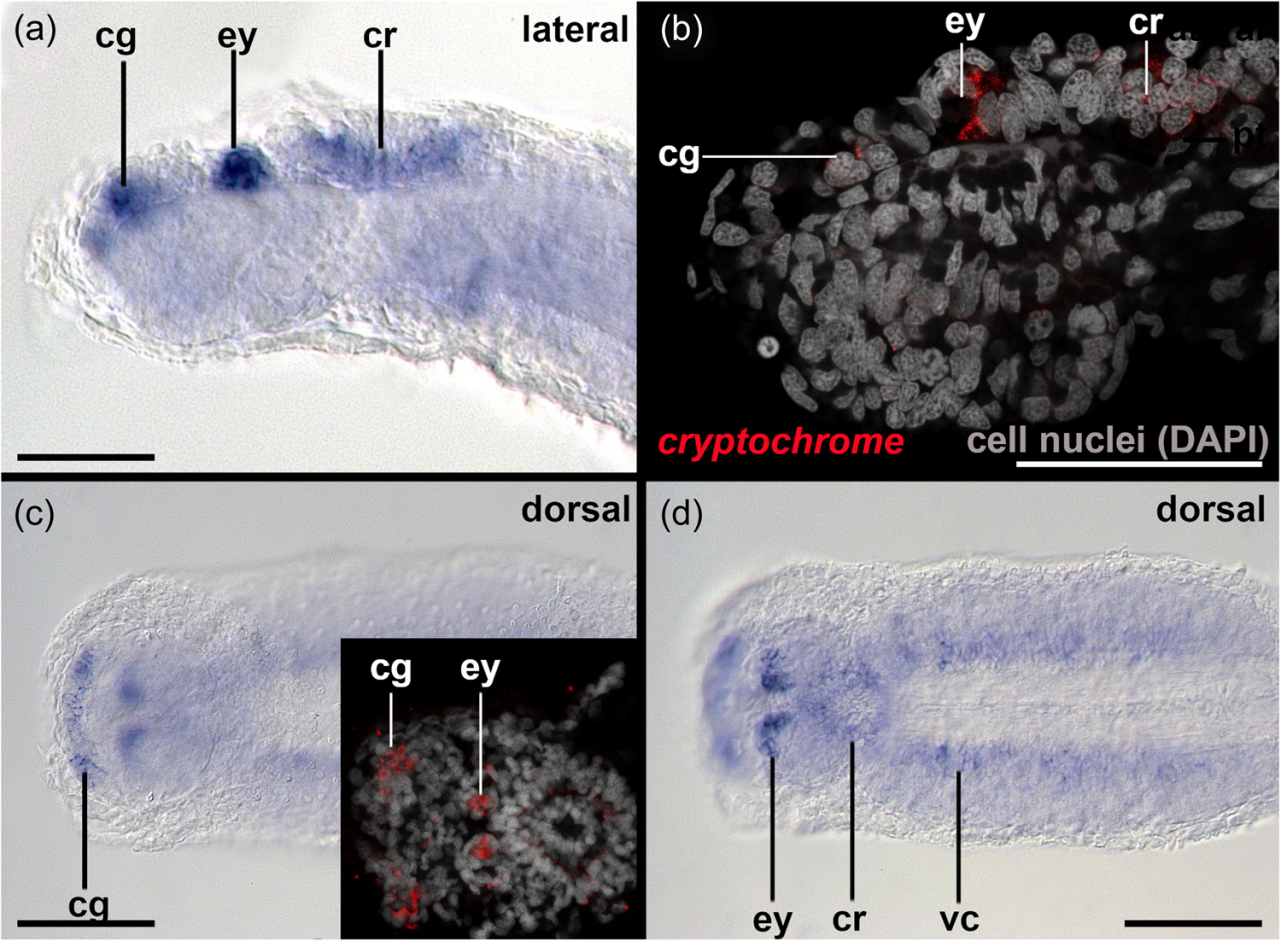
*Cryoptochrome* expression in early hatched juveniles of *Spadella cephaloptera* (~ 24 hph). Whole‐mount in situ hybridization of the head region, anterior to left. (a) *Cryoptochrome* is expressed in the region of the cerebral ganglia (cg), the eyes (ey), and the corona ciliata (cr) as highlighted by confocal laser scanning microscopy in (b). (c) Strong expression of *cryptochrome* in the cerebral ganglia and the proximal portions of the eyes. (d) *Cryptochrome* expression in the ventral nerve cords (vc). Scale bars: 100 µm.

Cells contributing to the eye anlagen of early encapsulated embryos of *S. cephaloptera* are present in the form of two nonepithelial clusters (see eye anlagen in Figures 2a and 3a–c). No pigment cell is present, although some photoreceptor cells already express *xenopsin1*. No *peropsin*+ and *cryoptochrome*+ cells are present in these early embryos (not shown). The eyes of young hatchlings are composed of cells that are epithelial and form a spherical shape that is partially filled with cells (Figure 2b). In hatchlings several *xenopsin1* + photoreceptor cells are located in the proximal and distal lateral regions but not in the anterior and posterior regions of the eyes (Figure 4a–d). During this stage *peropsin* is expressed in the cerebral ganglia and the perikaryal layers of the ventral nerve cord but not in the eyes (Figure 5a–d). *Cryptochrome* is expressed in photoreceptor cells that are located in the proximal and distal lateral portions of the eyes, in addition to the cerebral ganglia, the corona ciliata, and cells of the outer perikaryal layer of the ventral nerve cord (Figure 6a–d).

**Figure 7:**
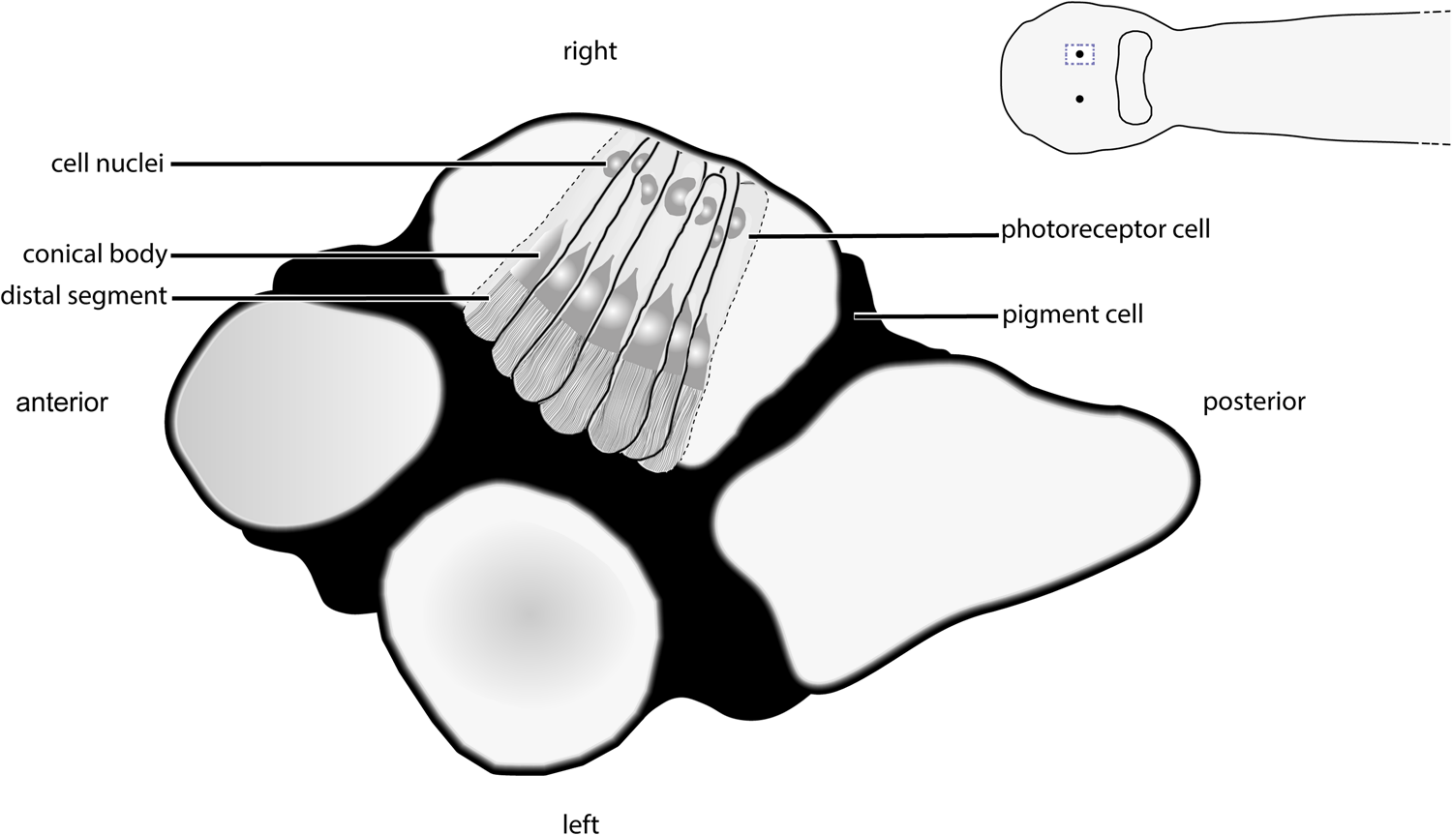
Adult eye anatomy of the chaetognath *Spadella cephaloptera* (dorsal view). The spherical‐shaped adult eyes exhibit photoreceptors with distal photoreceptive segments organized into four tightly packed domains. While the anterior and posterior domains are located more ventrally, the left and right domains are situated more dorsally. All four domains share and are separated by a single pigment cell. For clarity, only few photoreceptors of the right domain are shown. The three‐dimensional arrangement of the four domains was derived from confocal laser scanning experiments and certain details on the photoreceptors have been described previously (e.g., Shinn, 1997).

**Figure 8:**
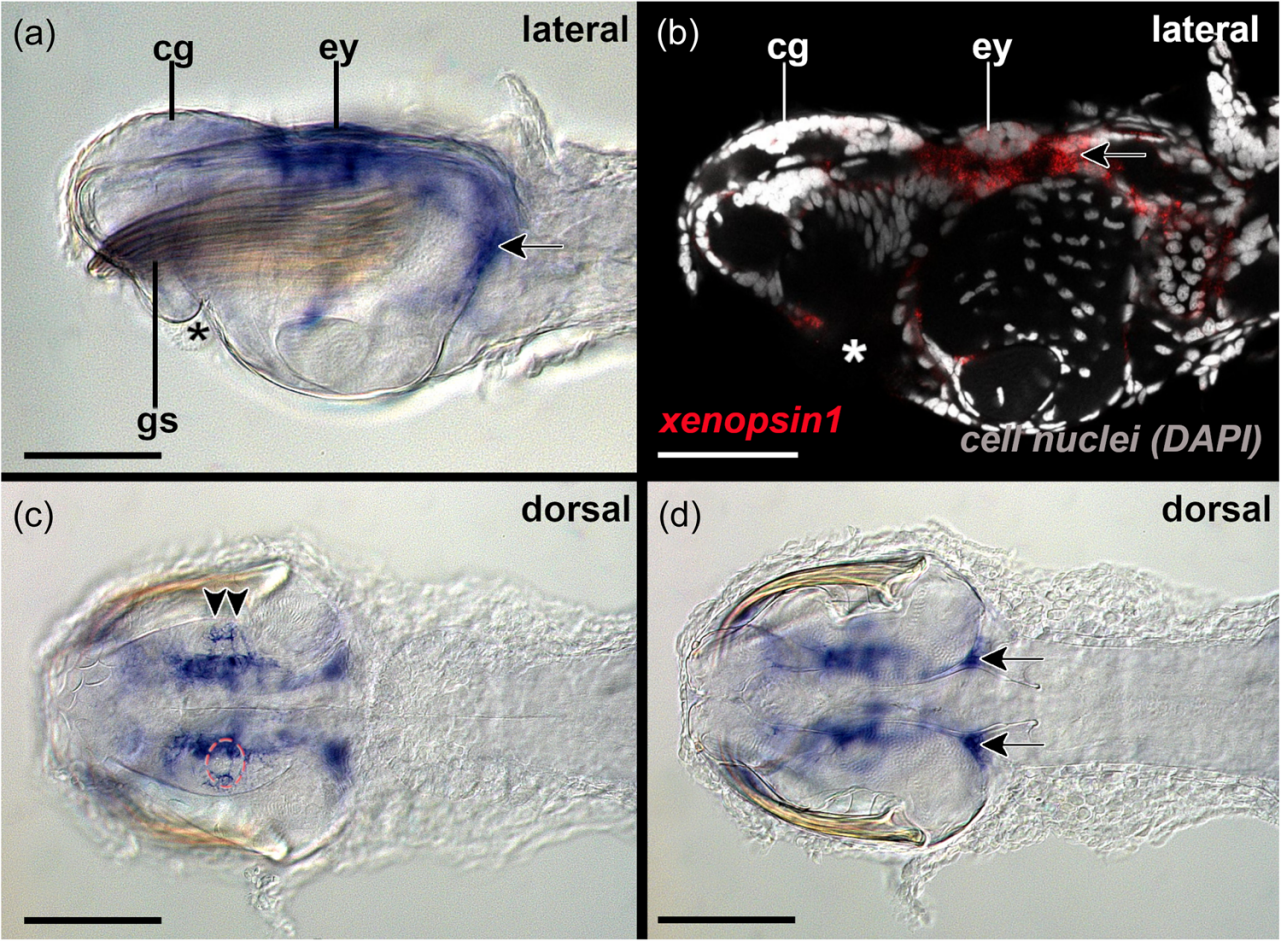
*Xenopsin1* expression in adults of *Spadella cephaloptera*. Whole‐mount in situ hybridization of the head region with anterior facing to the left. (a) *Xenopsin1* is expressed in the eyes (ey) and the region surrounding the latter. Unspecific staining marked with arrow. (b) Confocal laser scanning reflection scan highlights *xenopsin1* expression in the lateral eye and expression ventrally, anterior and posterior to it. (c) *Xenopsin1* is expressed in two photoreceptors (arrowheads) of each distolateral eye (ey) only. Stippled red circle highlights left eye. (d) Unspecific staining marked with arrows in ventroposterior head region. asterisk, mouth; cg, cerebral ganglion; gs, grasping spines. Scale bars: 200 µm.

**Figure 9:**
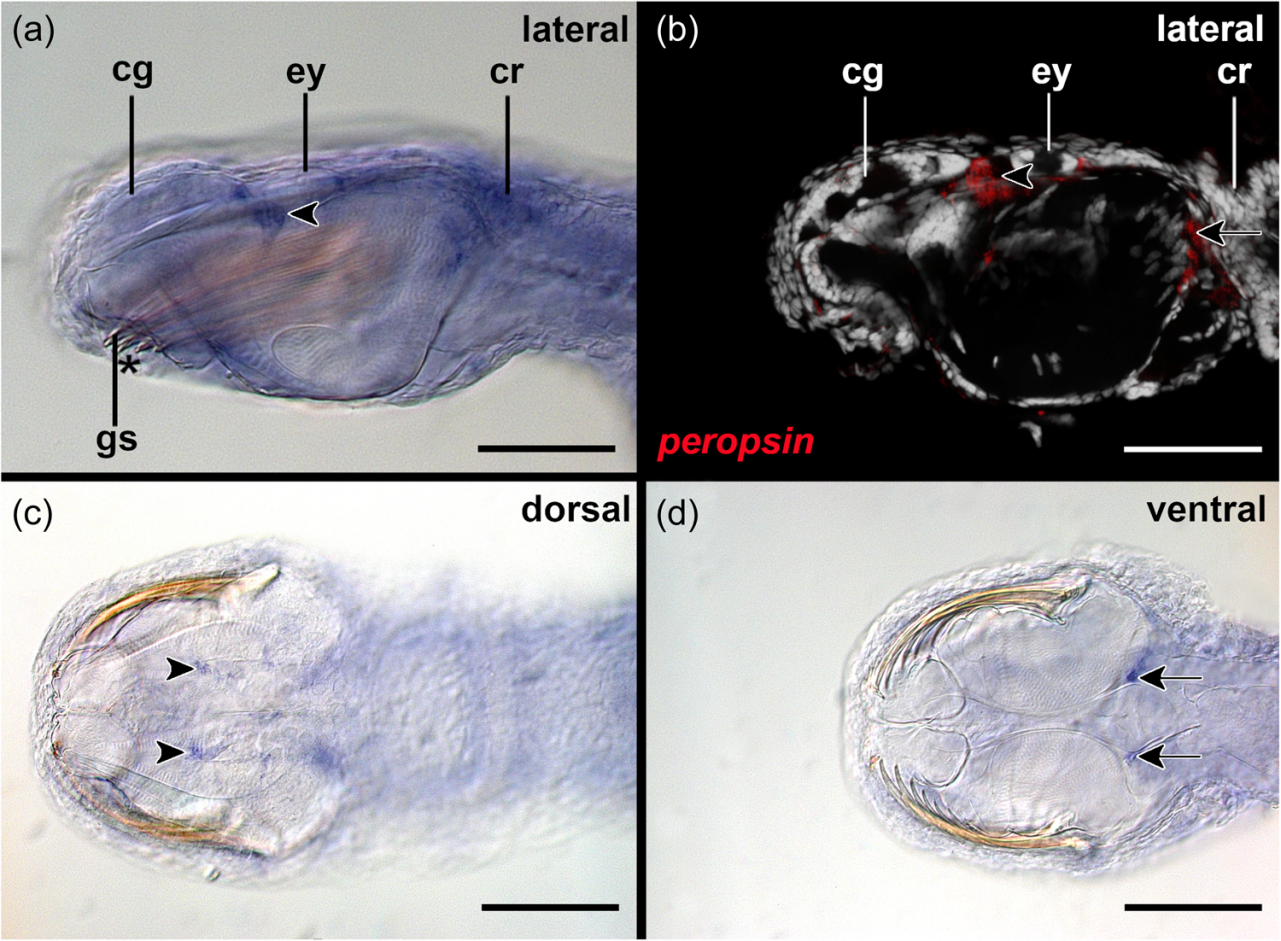
*Peropsin* expression in adults of *Spadella cephaloptera*. Whole‐mount in situ hybridization of the head region, anterior to the left. (a) *Peropsin* is expressed (arrowhead) antero‐ventrally to each eye (ey), and in the corona ciliata (cr). (b) *Peropsin* expression antero‐ventrally to the left eye highlighted by confocal laserscanning microscopy. (c) *Peropsin* expression antero‐ventrally to each eye and unspecifically stained structures (arrows; also shown in [d]). asterisk, mouth; cg, cerebral ganglion; cr, corona ciliata; gs, grasping spines. Scale bars: 200 µm.

**Figure 10:**
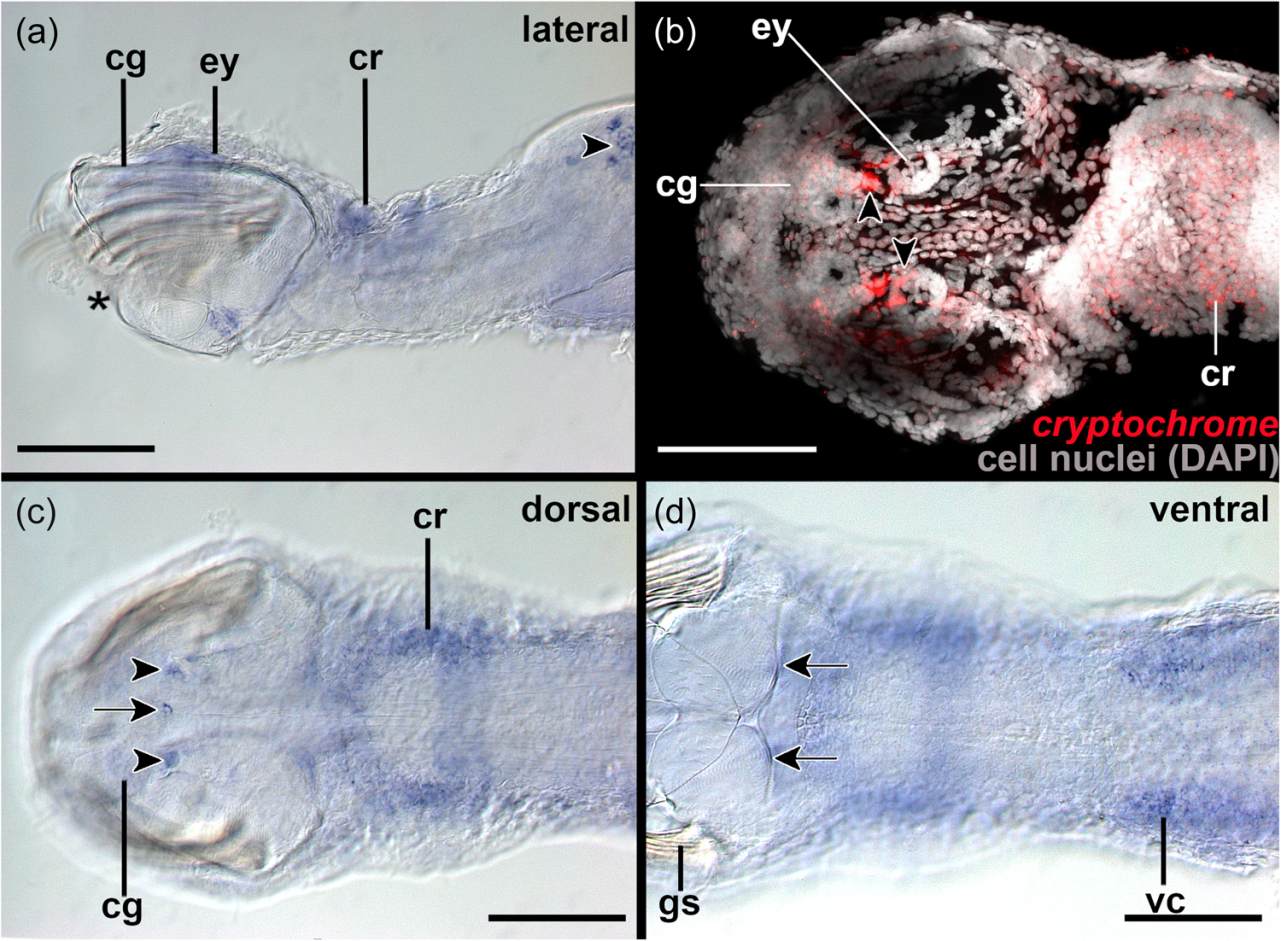
*Cryptochrome* expression in adults of *Spadella cephaloptera*. Whole‐mount in situ hybridization of the head region, anterior to the left. (a) *Cryptochrome* is expressed in the region of antero‐ventrally to each eye (eye) in this 2 weeks old juvenile. In addition, *cryptochrome* is expressed in the cerebral ganglia (cg), the corona ciliata (cr), and the few cells of the dorsal epidermis of the trunk. (b) Adults express *cryptochrome* in the same regions but not in the dorsal epidermis as highlighted by confocal laser scanning microscopy. The *cryptochrome* + cells are located anteroventrally to each eye (arrowheads). (c, d) An additional *cryptochrome* + cell (arrow) is located posterior to the retrocerebral pore, in the region between both above‐mentioned expression domains (arrowheads) and in the ventral nerve cord (vc) (arrow). Unspecifically stained structures (arrows in [d]). asterisk, mouth; gs, grasping spines. Scale bars: 200 µm.

The adult eyes are spherical in shape and composed of photoreceptors with distal photoreceptive segments that are arranged into four tightly packed domains (Figures 2c,d and 7; see figure 48 in Shinn, 1997). These four domains share, and are separated by, a single pigment cell: the two lateral domains are located dorsally, while the anterior (3) and posterior domains (4) are situated more ventrally (Figures 2c–e and 7). The optic nerves connect the eyes with cerebral ganglia (Figure 2c). *Xenopsin1* is only expressed in each of two photoreceptor cells of their distolateral eye and the region ventrally to the eye (Figure 8a–d). Adults express *peropsin* in the region antero‐ventrally to each eye and faintly in the corona ciliata (Figure 9a–d). In 2‐week‐old juveniles, *cryptochrome* is expressed in cells of the cerebral ganglia, in cells of the corona ciliata, the ventral nerve cord, and in few cells of the dorsal trunk epidermis (Figure 10a). In addition, *cryptochrome* is expressed anteroventral to the eyes in each two adjacent cell somata, and in two cell somata posteroventrally to the retrocerebral pore (Figure 10c). Adults express *cryptochrome* in the same regions but not in the dorsal epidermis (Figure 10c,d). An additional *cryptochrome* + cell is located in the region between both above‐mentioned expression domains antero‐ventrally to the eyes and in the ventral nerve cord (Figure 10b–d). Unspecific staining in the ventroposterior adult head region was observed in expression patterns of all three above‐mentioned genes and in yet unpublished expression patterns of other genes (arrows in Figures 8d, 9d, and 10d).

## 4 DISCUSSION

### 4.1 Ocular photoreceptors of chaetognaths express probably only a single opsin

The existence of numerous elaborate photoreceptors in the eyes suggests that vision plays an important role during predation and escape responses of chaetognaths (Eakin & Westfall, 1964; Goto & Yoshida, 1984). Our thorough screen of a transcriptome derived from total RNA of various developmental stages and adults of *S. cephaloptera* however only yielded two copies of *sce‐xenopsin* and a single copy of *sce‐peropsin*. Note that also two copies of *xenopsin* have been identified for another chaetognath species, *Pterosagitta draco* (Rawlinson et al., 2019). Our subsequent gene expression analysis demonstrated that *sce‐xenopsin1* but not *sce‐peropsin* is expressed in the eyes of developmental stages and adults of the chaetognath *S. cephaloptera* (Figures 3, 4, 5). In early embryos and hatchlings *xenopsin1* + photoreceptors are located in the lateral portions of the eyes (Figure 4c,d), raising the questions of whether the anterior and posterior cells of the eyes are indeed photoreceptors and if so, which opsin is expressed in the latter. To answer these questions, an intensive search for other light‐sensitive proteins identified an ortholog of *cryptochrome*. *Sce‐cryptochrome* is expressed in fewer cells than *sce‐xenopsin1* but in the same lateral domains in hatchlings (although not in adults) (Figures 4, 6, 8, and 10). Putative co‐expression of *sce‐xenopsin1* and *sce‐cryptochrome* in the lateral photoreceptors of the chaetognath eyes is reminiscent of the condition in the annelid *Platynereis dumerilii* in which *cryptochrome* and *c‐opsin* are co‐expressed in the apical nervous system (Tosches et al., 2014).

### 4.2 Nonvisual light‐sensitive cells in the chaetognath nervous system?

*Sce‐cryptochrome* and *sce‐peropsin* are both expressed in the dorsal cerebral ganglia and the ventral nerve cord, a condition that resembles the co‐expression of *cryptochrome* and *peropsin* in the apical nervous system of the annelid *Platynereis dumerilii* (Tosches et al., 2014). These findings may indicate that the dorsal cerebral ganglia and portions of the ventral nerve cord are domains of nonvisual light sensitivity as has been proposed for *P. dumerilii*, in which light detection, circadian entrainment, and melatonin release are performed by a single photoreceptive zone (Tosches et al., 2014).

Many different roles have been proposed for the corona ciliata including excretory (chaetognaths lack nephridia), (chemo)sensory, or secretory (glandular) function (Harzsch et al., 2015; Müller et al., 2014; Shinn, 1997). Our study demonstrates that *sce‐cryptochrome* is expressed in distal and proximal cells of the corona ciliata suggesting that cells of this organ may be involved in circadian entrainment (Figure 10a–c).

### 4.3 The Spiralia—A clade unveiling tremendous photoreceptor diversity

Recent studies revealed diverse combinations of photoreceptors and opsins within the Spiralia (Döring et al., 2020; Passamaneck et al., 2011; Rawlinson et al., 2019; Vöcking et al., 2017). The trochophore larva of the polyplacophoran mollusk *Leptochiton asellus* exhibits multiciliated, microvillar photoreceptors that co‐express *xenopsin* and *r‐opsin* (Vöcking et al., 2017), while the larva of the brachiopod *Terebratalia transversa* exhibits eyespots with ciliated photoreceptors expressing *xenopsin* (Passamaneck et al., 2011). A recent study on the platyhelminth *Maritigrella crozieri* showed *xenopsin* expression in the larval epidermal eye, in the cerebral eyes, and in the adult phaosomal photoreceptors, which all consist of ciliated photoreceptors (Rawlinson et al., 2019). In the larval epidermal eye of *M. crozieri*, a single photoreceptor cell produces many cilia which form a lamella packed into a pigmented pocket formed of an adjacent cell, similar to how the array of chaetognath ciliated photoreceptors jut into one of several pockets formed in a central pigment cell (Rawlinson et al., 2019; Figure 7). Cilia of the chaetognath photoreceptors elaborate into annulated lamellae within these pockets (Goto et al., 1984). While the chaetognath eye is not phaosomous as described earlier (Purschke et al., 2006), it is encapsulated by the extracellular matrix and sheath cells as are the phaosome and epidermal eye of the platyhelminth *M. crozieri* (Rawlinson et al., 2019; Figure 7. It has been speculated that the phaosomal photoreceptive structures of platyhelminths are evolutionarily derived (Sopott‐Ehlers et al., 2001), however, they are also present among annelids and phaosome‐bearing annelids have not been exhaustively searched for *xenopsin* orthologs yet. Hence, it is tempting to speculate that each species‐specific phaosome‐like structure may be derived from a ciliated photoreceptor expressing *xenopsin* that was present in the last common ancestor of Spiralia. Together, our and other comparative studies emphasize the importance to investigate taxa such as Gnathifera to unravel the evolution of spiralian and bilaterian body plans.

## AUTHOR CONTRIBUTIONS

Tim Wollesen designed the study, raised the chaetognaths, extracted the RNA, and carried out all experiments. Sonia V. Rodriguez Monje and Tim Wollesen assembled the transcriptome and carried out the phylogenetic analyses. Tim Wollesen drafted the manuscript and Sonia V. Rodriguez Monje, Adam P. Oel, and Detlev Arendt commented on the manuscript. All authors read and approved a final version of the manuscript.

## CONFLICT OF INTEREST STATEMENT

The authors declare no conflict of interest.

### PEER REVIEW

The peer review history for this article is available at https://www.webofscience.com/api/gateway/wos/peer-review/10.1002/jez.b.23193.

## Supporting information

Supplementary information.

## Data Availability

All sequences of *Spadella cephaloptera* analyzed in this study have been published on GenBank and the raw reads of the transcriptome have been deposited on SRA (https://www.ncbi.nlm.nih.gov/sra). All sequences of *S. cephaloptera* analyzed in this study have been published on GenBank and the raw reads of the transcriptome have been deposited on Zenodo (https://zenodo.org/record/7602960#.Y90U0oSZOUk/DOI:10.5281/zenodo.7602960).
