# Supplementary material for: Characterization of eyes, photoreceptors, and opsins in developmental stages of the arrow worm Spadella cephaloptera (Chaetognatha)
Source: J Exp Zool B Mol Dev Evol. 2023 Feb 28;340(5):342–53. doi: 10.1002/jez.b.23193 (PMC10952353; doi:10.1002/jez.b.23193)
Supplement: Supplementary file 1 — Supplementary information. [file JEZ-340-342-s001.docx]

**Supplementary Material:**

**Characterization of eyes, photoreceptors and opsins in developmental stages of the arrow worm *Spadella cephaloptera* (Chaetognatha)**

**Running title: chaetognath eye development**

**Tim Wollesen^a*^**, Sonia Victoria Rodríguez Monje^a^, Adam Phillip Oel^b^, and Detlev Arendt^b^

**
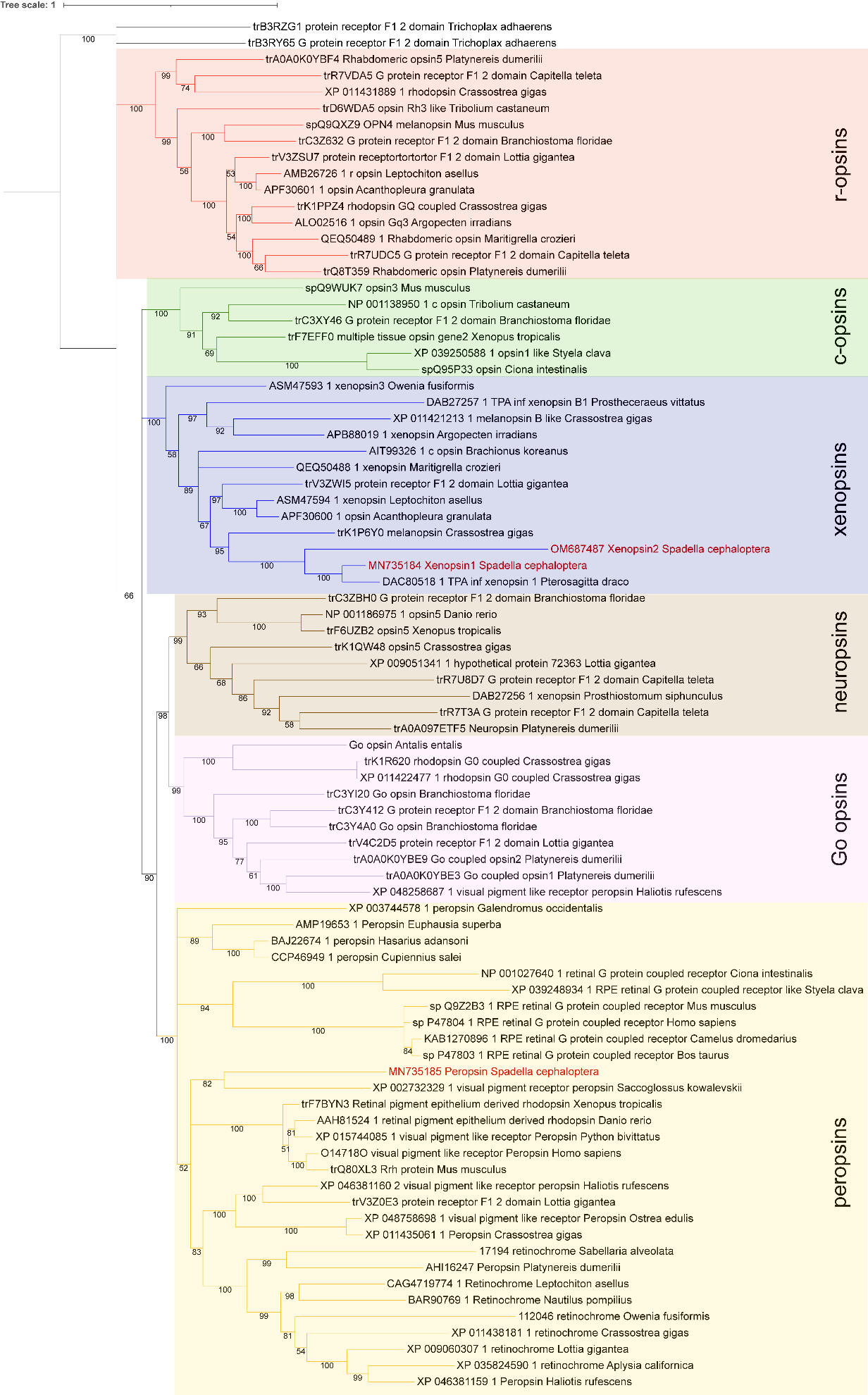
**

**Supplementary FIGURE 1** Phylogenetic tree of opsins. Majority consensus phylogenetic tree based on Bayesian analysis. Bootstrap values are shown. Xenopsin and Peropsin sequences of the chaetognath *Spadella cephaloptera* are highlighted in red. Nomenclature follows Ramirez et al. 2016 and Vöcking et al. 2021. The Placopsins of *Trichoplax adhaerens* served as outgroups.


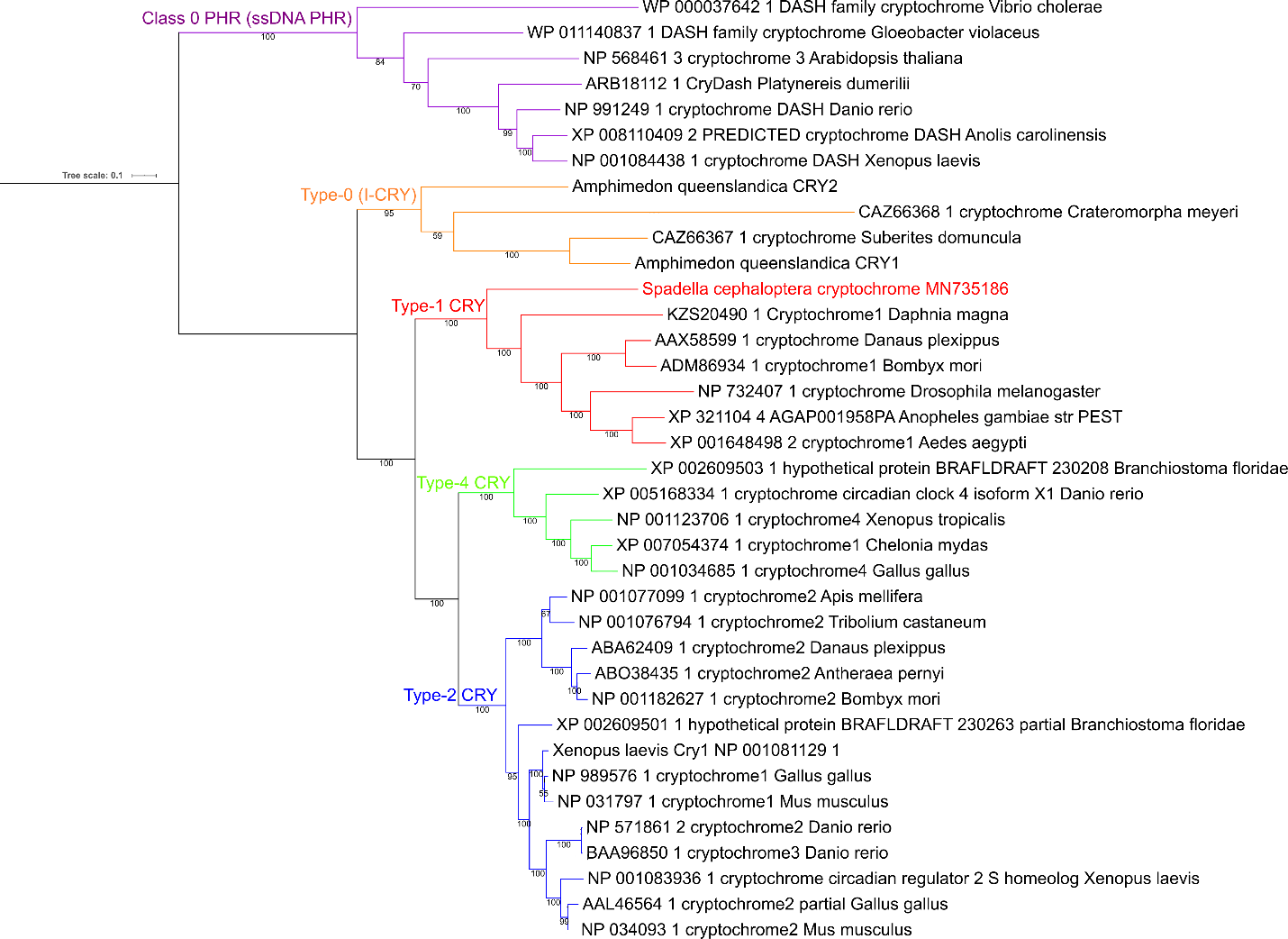


**Supplementary FIGURE 2** Phylogenetic tree of cryptochromes. Majority consensus phylogenetic tree based on Bayesian analysis. Bootstrap values are shown. The Cryptochrome of the chaetognath *Spadella cephaloptera* is highlighted in red. Nomenclature follows Ozturk (2017). Class 0 PHR (ssDNA PHR) cryptochromes serve as an outgroup.
